# Supplementary material for: Perfluorocarbons cause thrombocytopenia, changes in RBC morphology and death in a baboon model of systemic inflammation
Source: PLoS One. 2022 Dec 30;17(12):e0279694. doi: 10.1371/journal.pone.0279694 (PMC9803179; doi:10.1371/journal.pone.0279694)
Supplement: S1 File — (PDF) [file pone.0279694.s001.pdf]

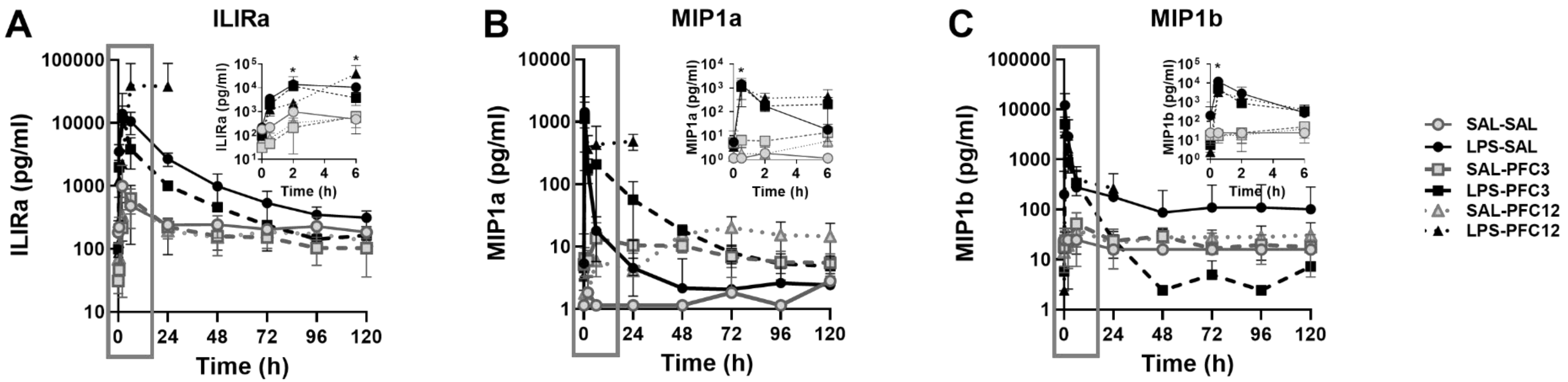

S1Fig. Baboons treated with an infusion of LPS in the presence or absence of PFC show an immediate increase in ILIRa (A), MIP1a (B) and MIP1b (C). Inset panels show early time points on an expanded scale. Open or closed symbols depict initial infusion conditions with open for saline and closed for LPS. Graphs of initial infusions with saline are in grey. Symbol type depicts second infusion with squares, circles and triangles for saline, PFC3 and PFC12, respectively. Error bars depict SEM. Data that are significantly different, $p<0.05$, are shown compared to: baseline, by *. Both treatment group and time show significant differences with an interaction between both treatment group and time.

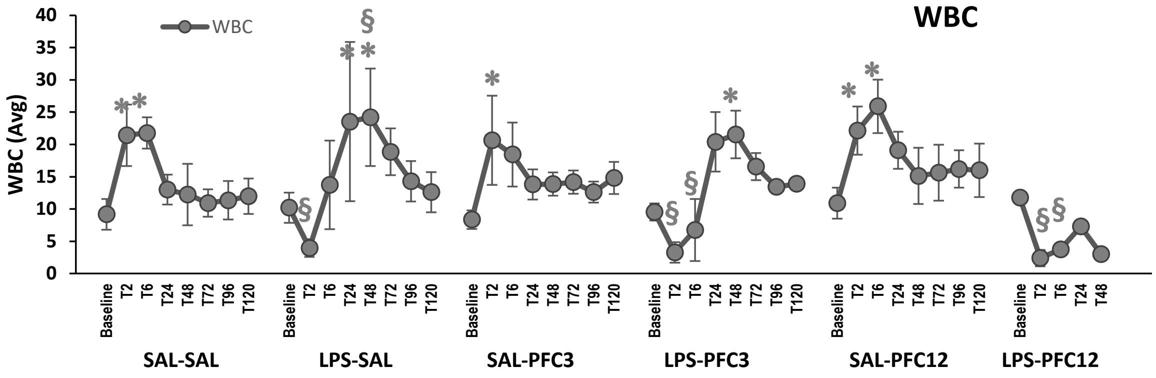

S2 Fig. White blood respond to saline in the presence or absence of PFC with an immediate increase; white blood cell respond to LPS with an initial decrease and then subsequent increase in WBC numbers. Error bars depict SEM. Data that are significantly different, p<0.05, are shown compared to: baseline, by *; to SAL-SAL value, by §. Both treatment group and time show significant differences with an interaction between both treatment group and time.

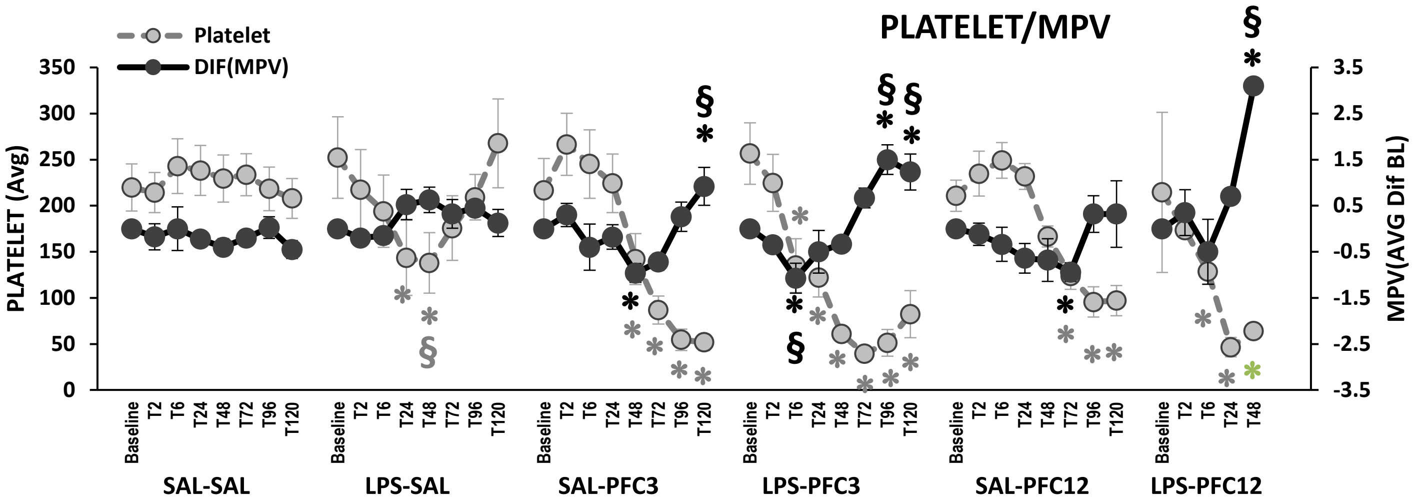

S3 Fig. PFC addition exacerbates LPS induced thrombocytopenia; mean platelet volume (mpv; difference from baseline values) decreases with LPS and/or PFC infusion. As platelet numbers recover, mpv increases. Platelet number as shown in Figure 3 is re-graphed here on the left axis (blue). MPV (difference from baseline values) is graphed on the right axis (green).
Error bars depict SEM. Data that are significantly different, p<0.05, are shown compared to: baseline, by *; to SAL-SAL value, by §. Both treatment group and time show significant differences with an interaction between both treatment group and time for platelet and MPV.

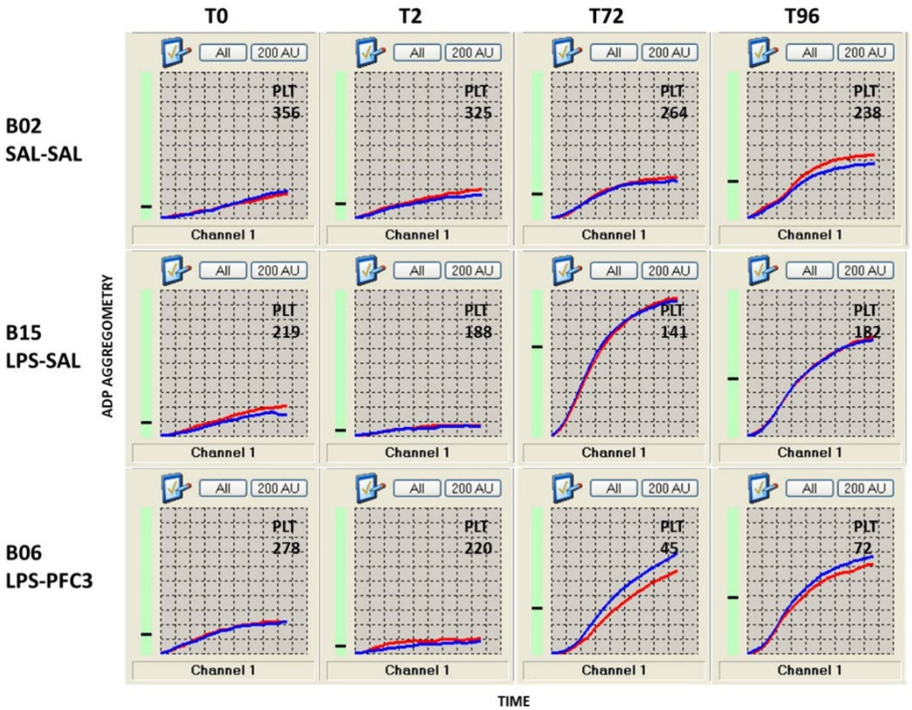

S4 Fig. Representative curves of platelet aggregation. Multiplate screen shots of the response to ADP stimulation from three subjects over the time course studied are shown. Three study groups only are given. Platelet values at each time point are shown in the upper left of the data.

Supplemental Figures- S5-S16
TEM All groups T24 whole blood &
Necropsy Results

S5 Fig. Representative images from TEM of all groups at 24h.

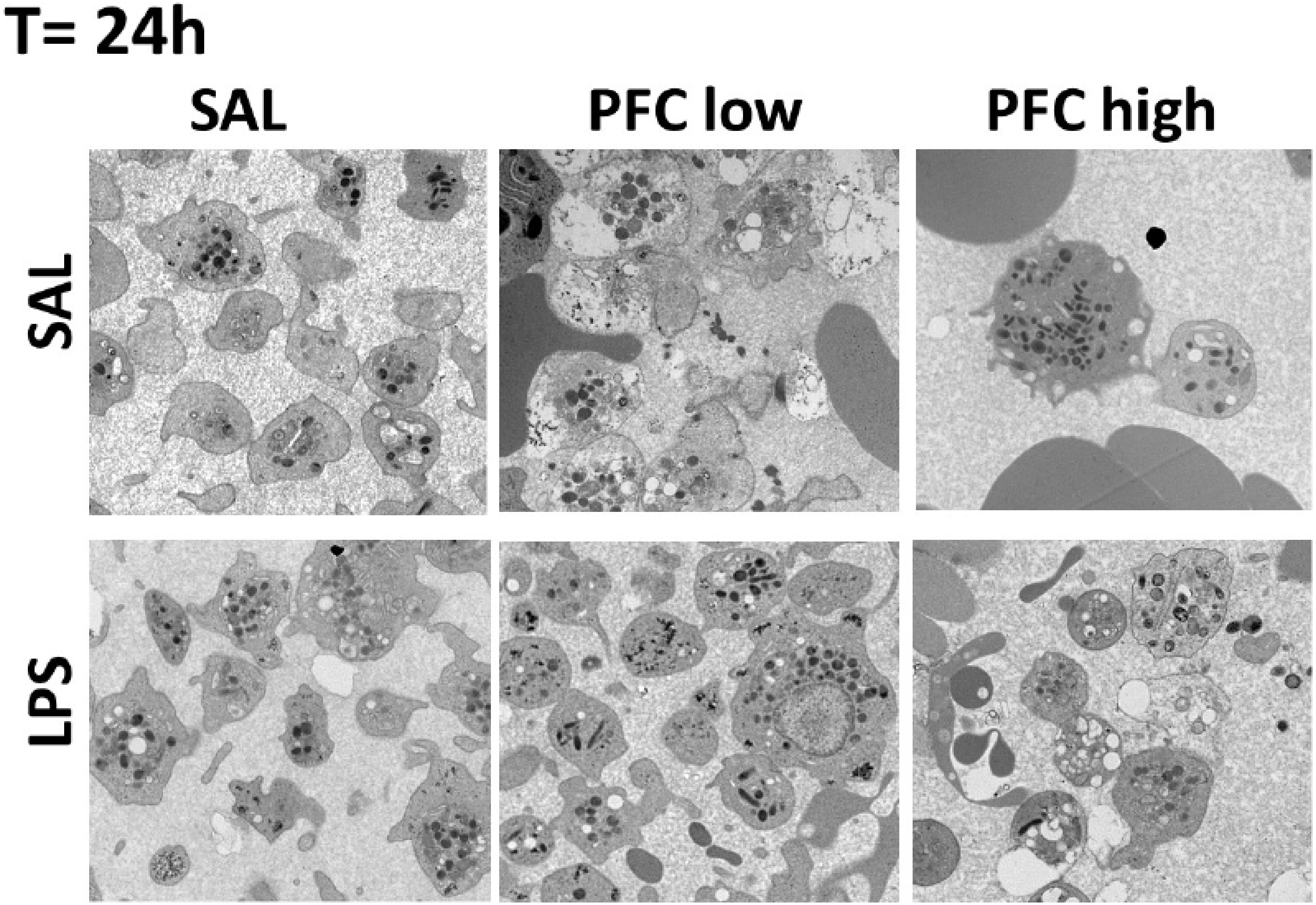

S6 Fig. Representative images from TEM of group 1, SAL/SAL. The bar represents 2 microns.

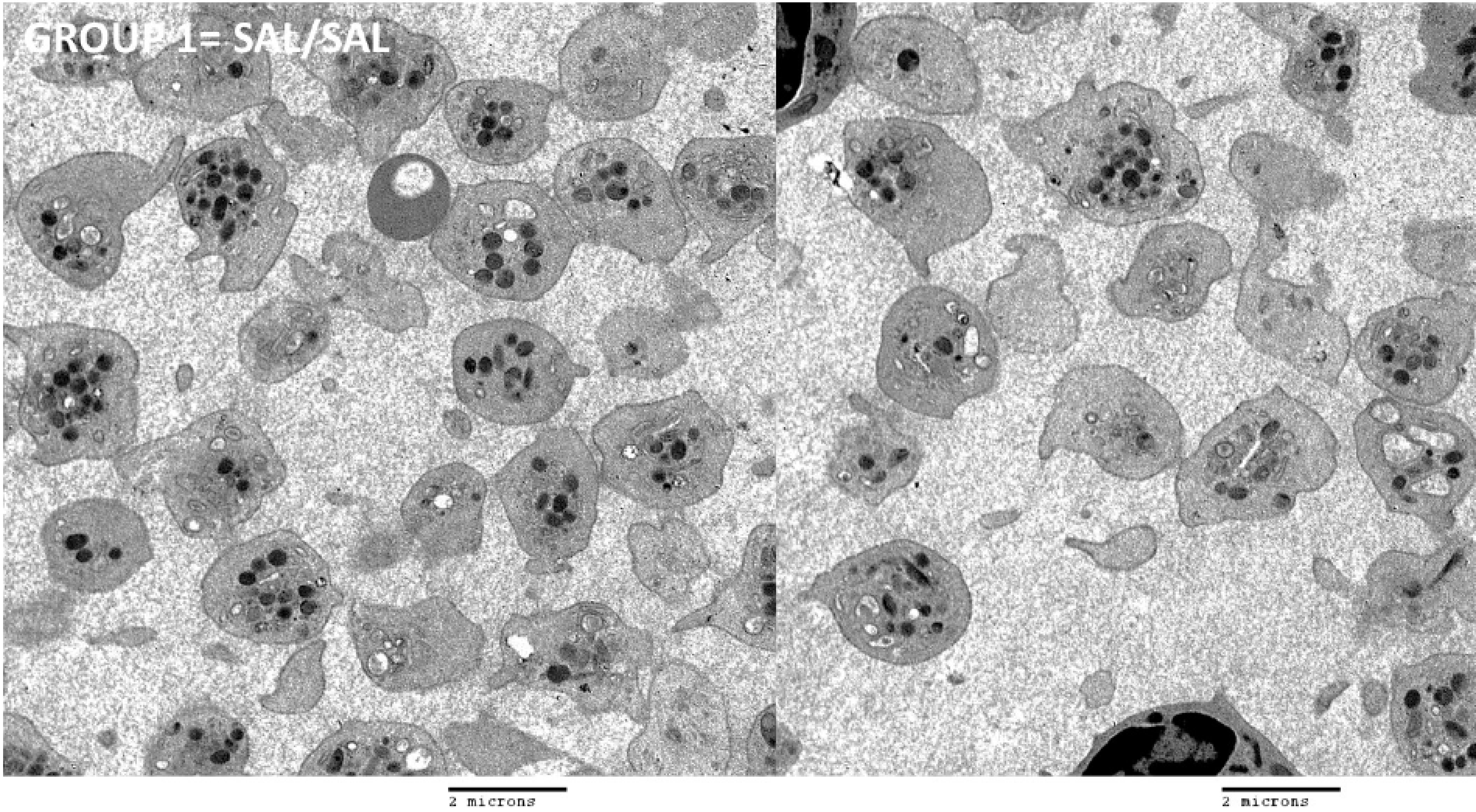

SAL-SAL, 24h

S7 Fig. Representative images from TEM of group 2, LPS/SAL. The bar represents 2 microns.

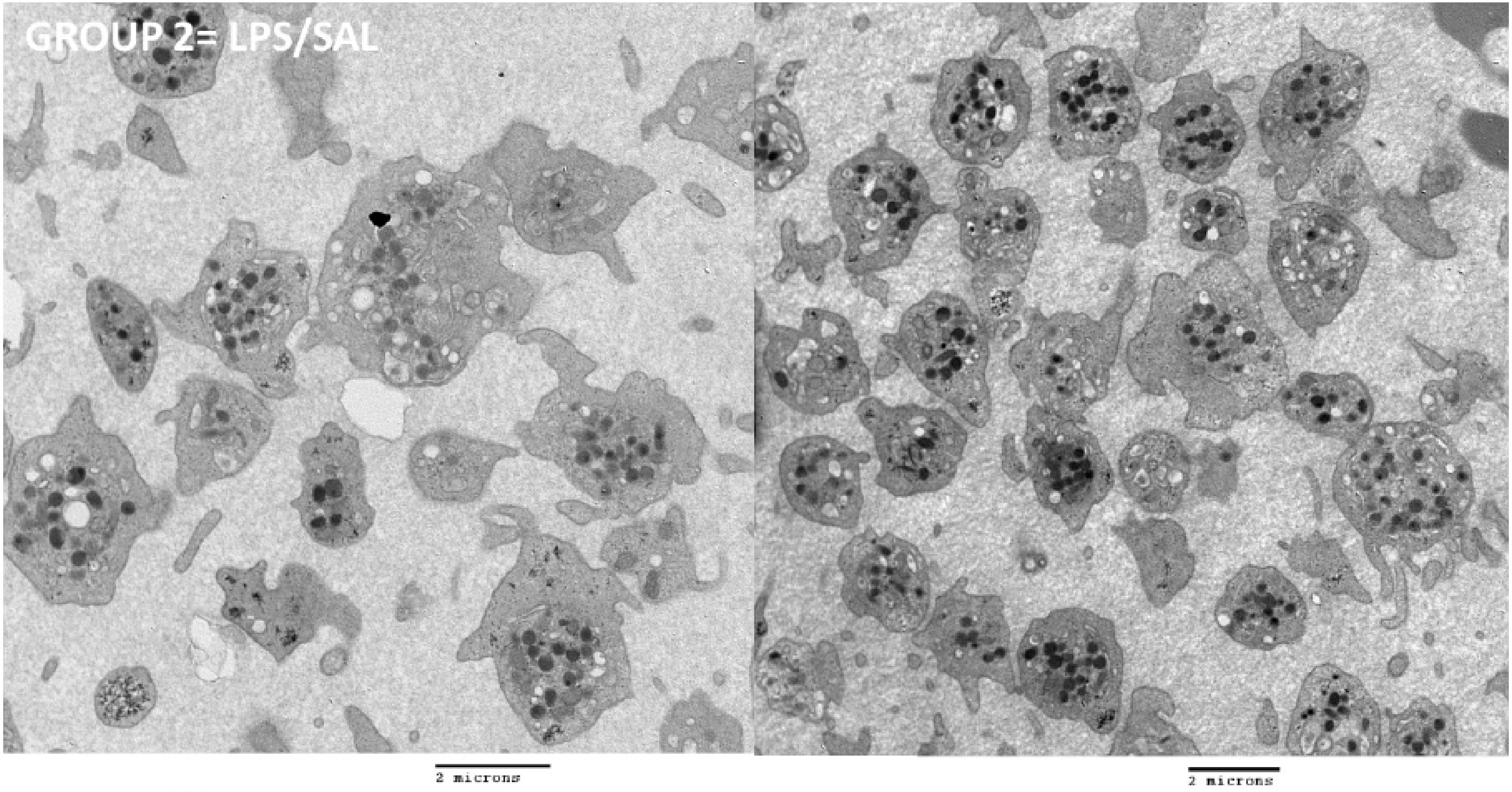

LPS-SAL, 24h

S8 Fig. Representative images from TEM of group 3, SAL/PFC3. The bar represents 2 microns.

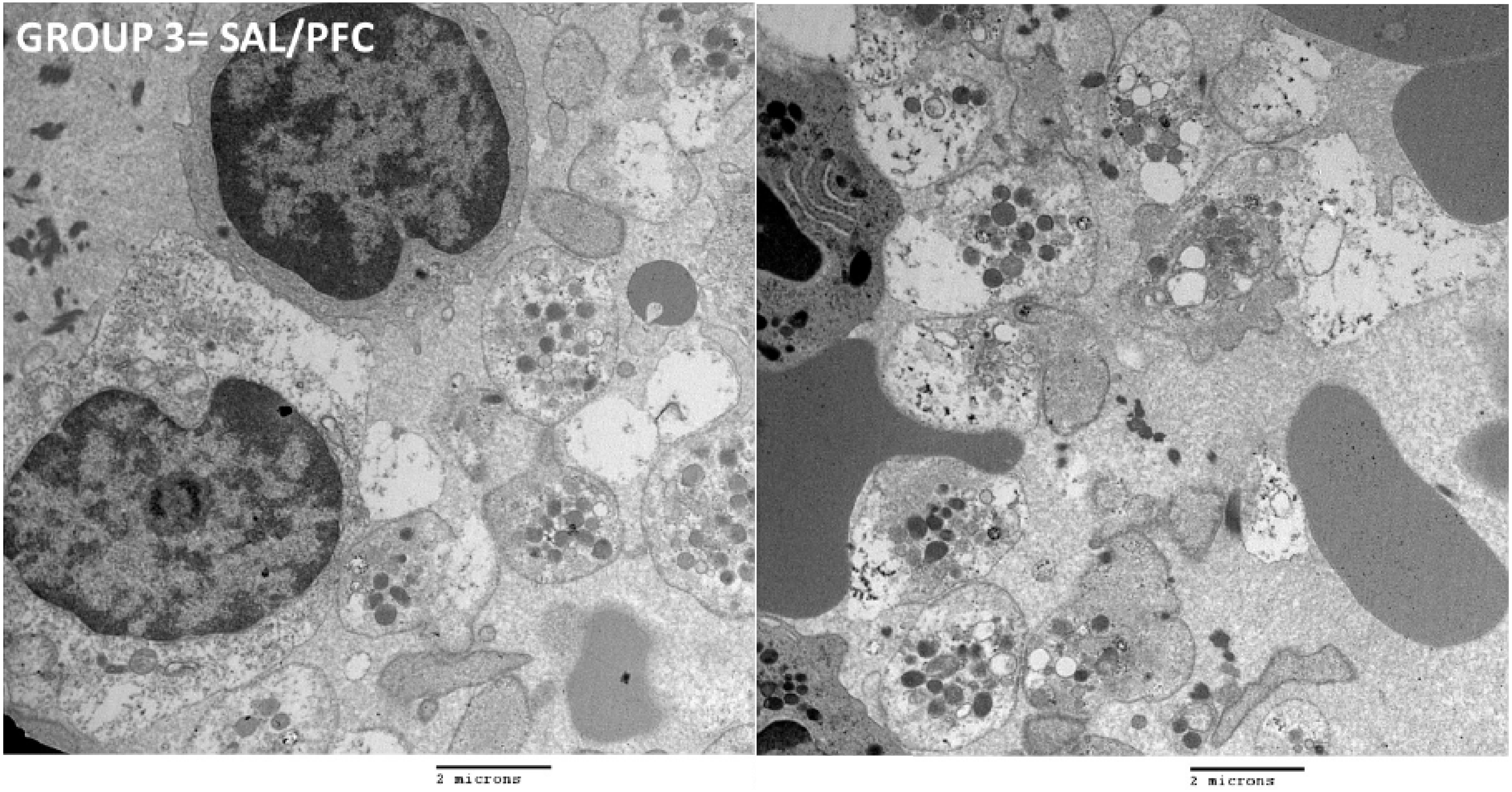

SAL-PFC3, 24h

S9 Fig. Representative images from TEM of group 4, LPS/PFC3. The bar represents 2 microns.

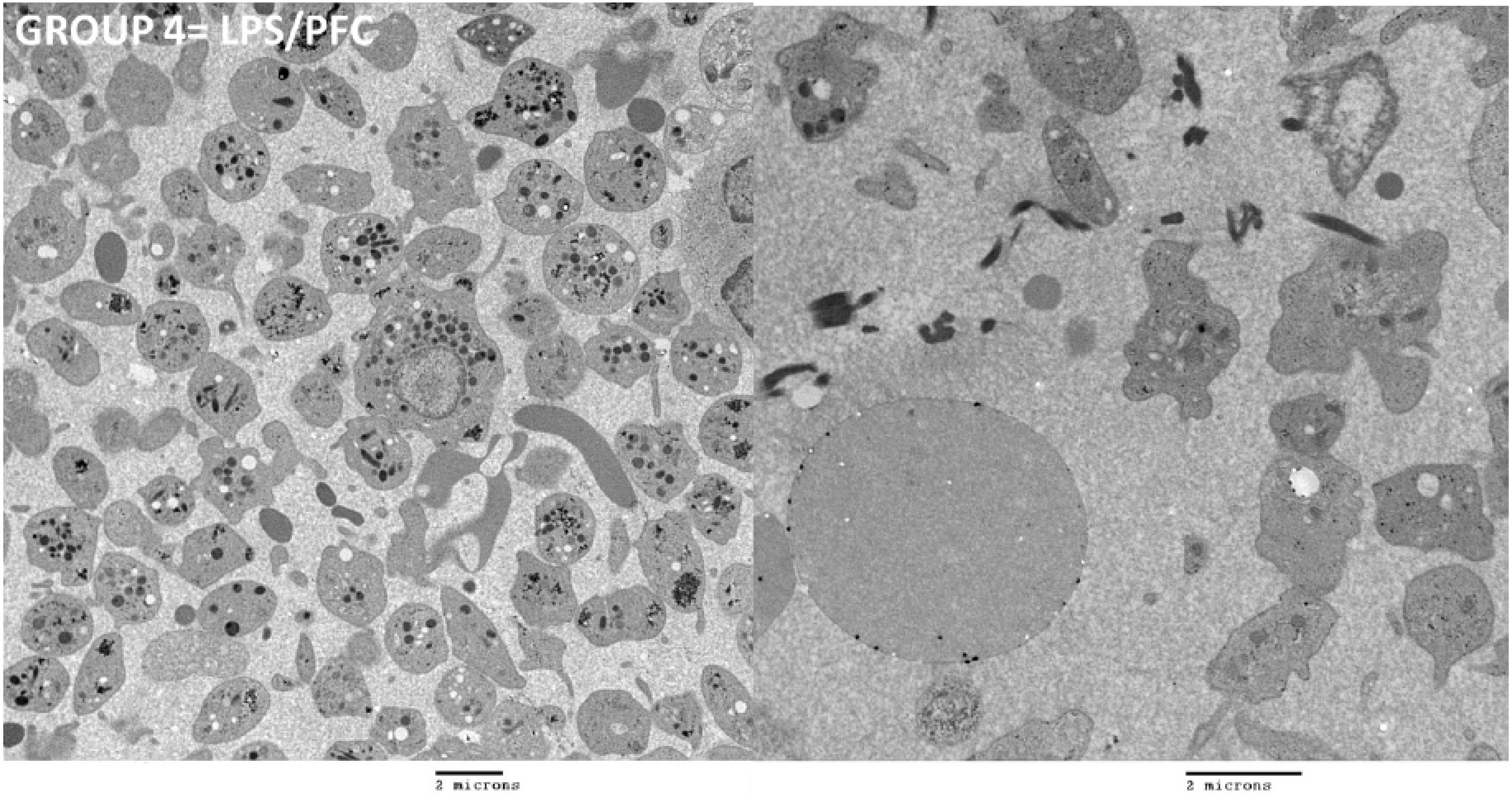

LPS-PFC3, 24h

S10 Fig. Representative images from TEM of group 5, SAL/PFC12. The bar represents 2 microns.

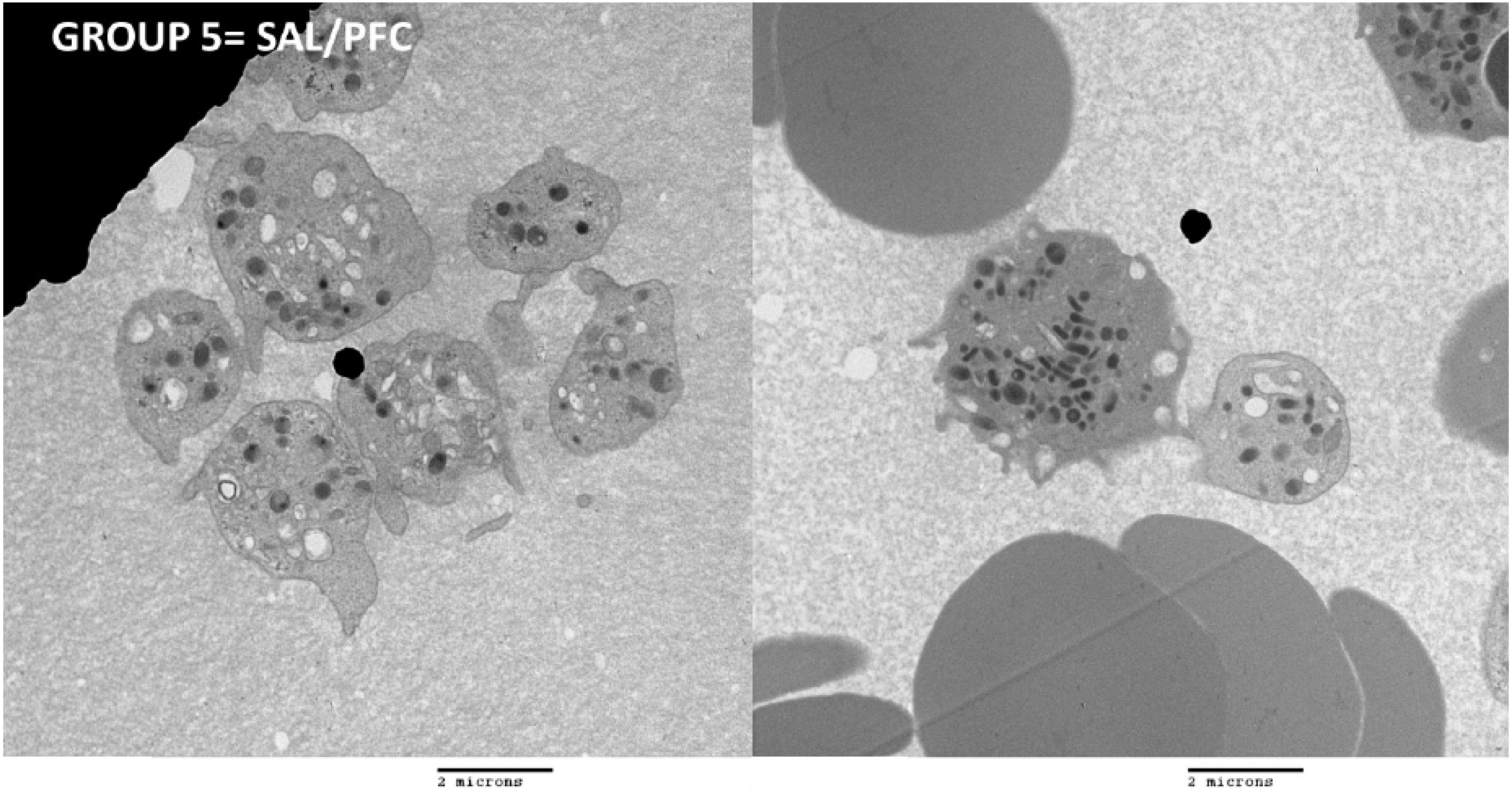

SAL-PFC12, 24h

S11 Fig. Representative images from TEM of Baboon 1, Group 6, SAL/PFC12. The bar represents 2 microns.

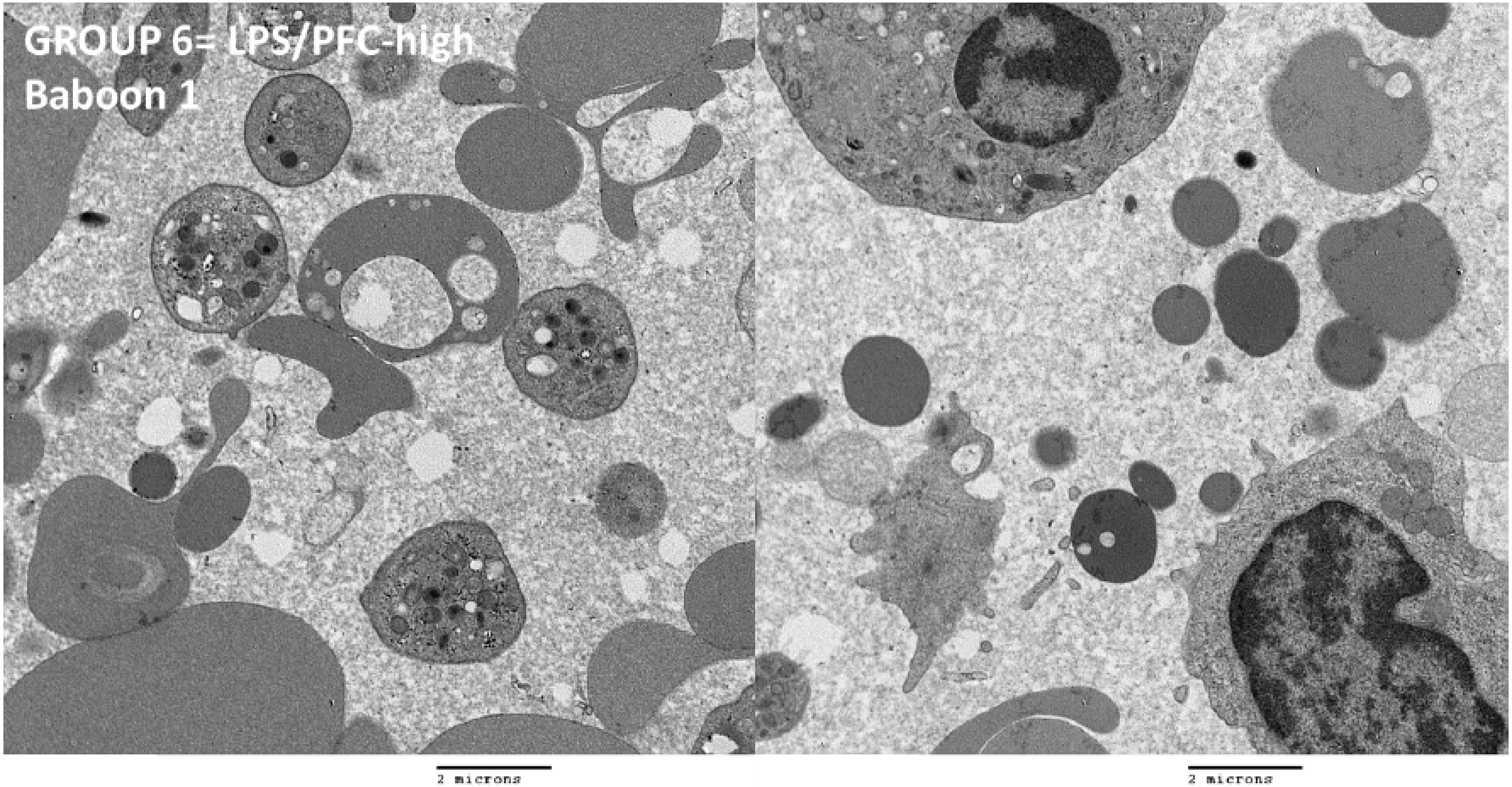

LPS-PFC12, 24h

S12 Fig. Representative images from TEM of Baboon 2, Group 6, SAL/PFC12. The bar represents 2 microns.

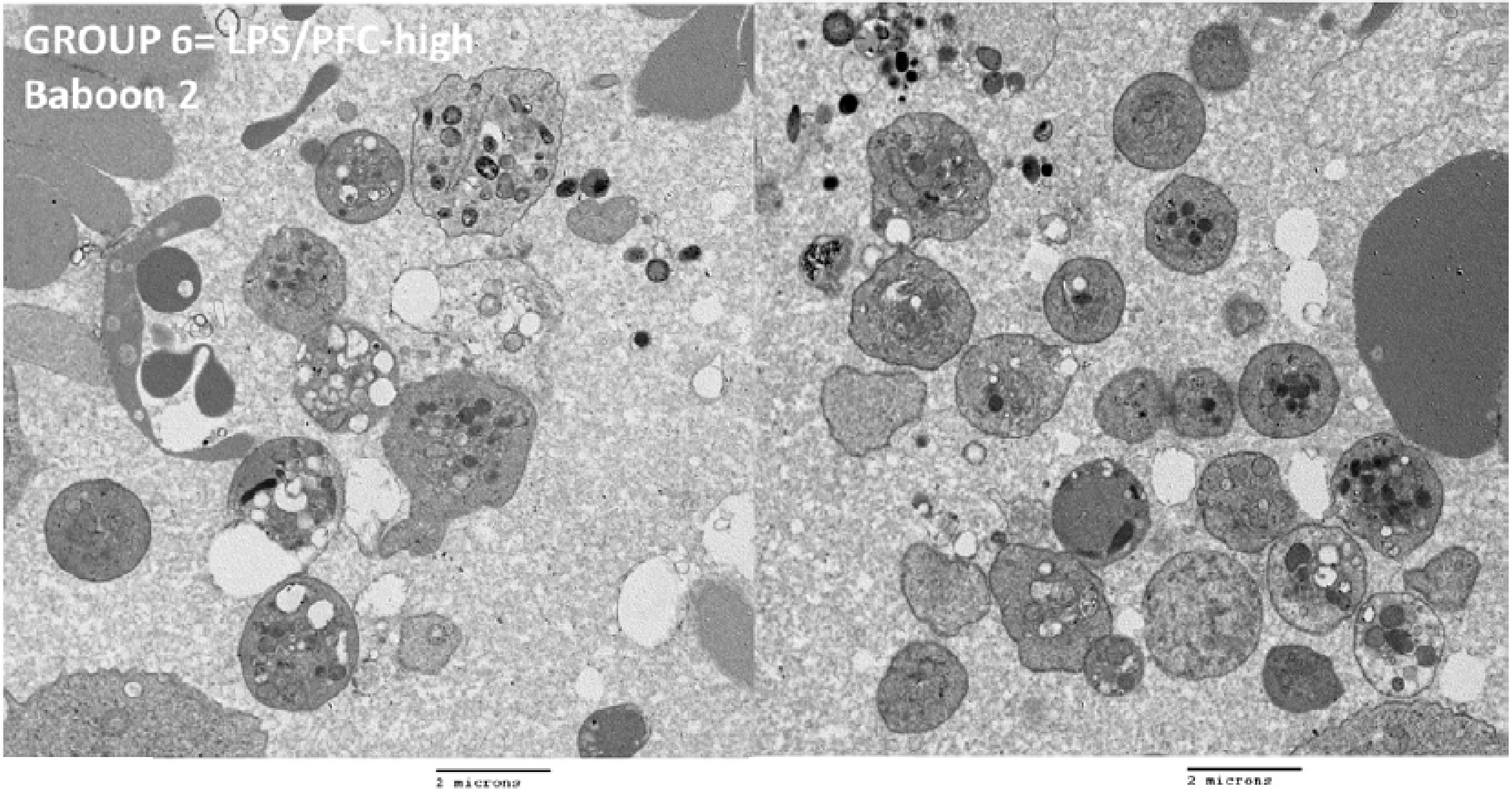

LPS-PFC12, 24h

S13 Fig. Representative images from TEM of Baboon 2, Group 6, SAL/PFC12. The bar represents 2 microns and 500 nm.

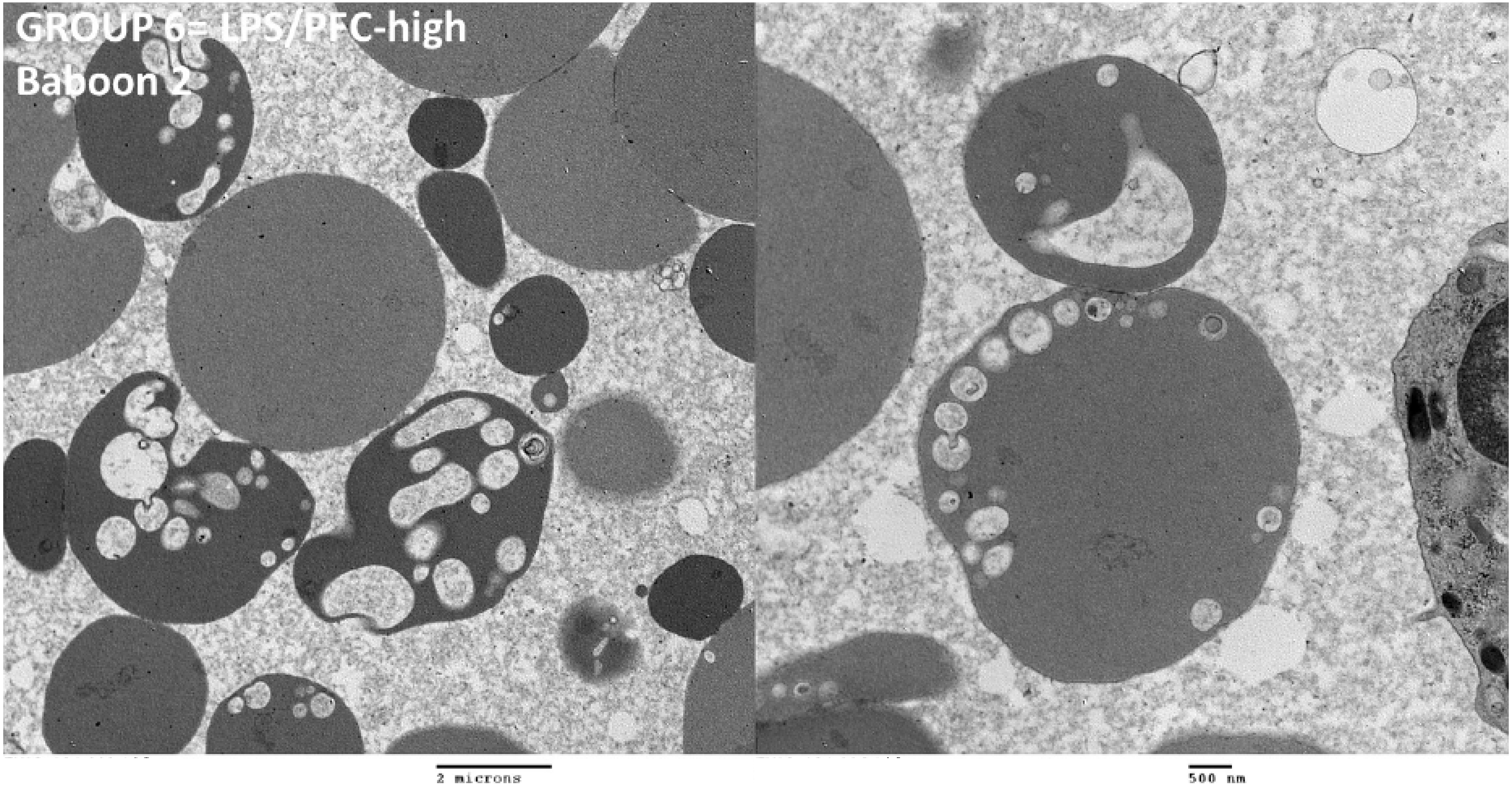

LPS-PFC12, 24h

S14 Fig. Representative images from TEM of necropsy of Baboon 1, Group 6, SAL/PFC12. Kidney is shown.

NECROPSY RESULTS: Kidney TEM

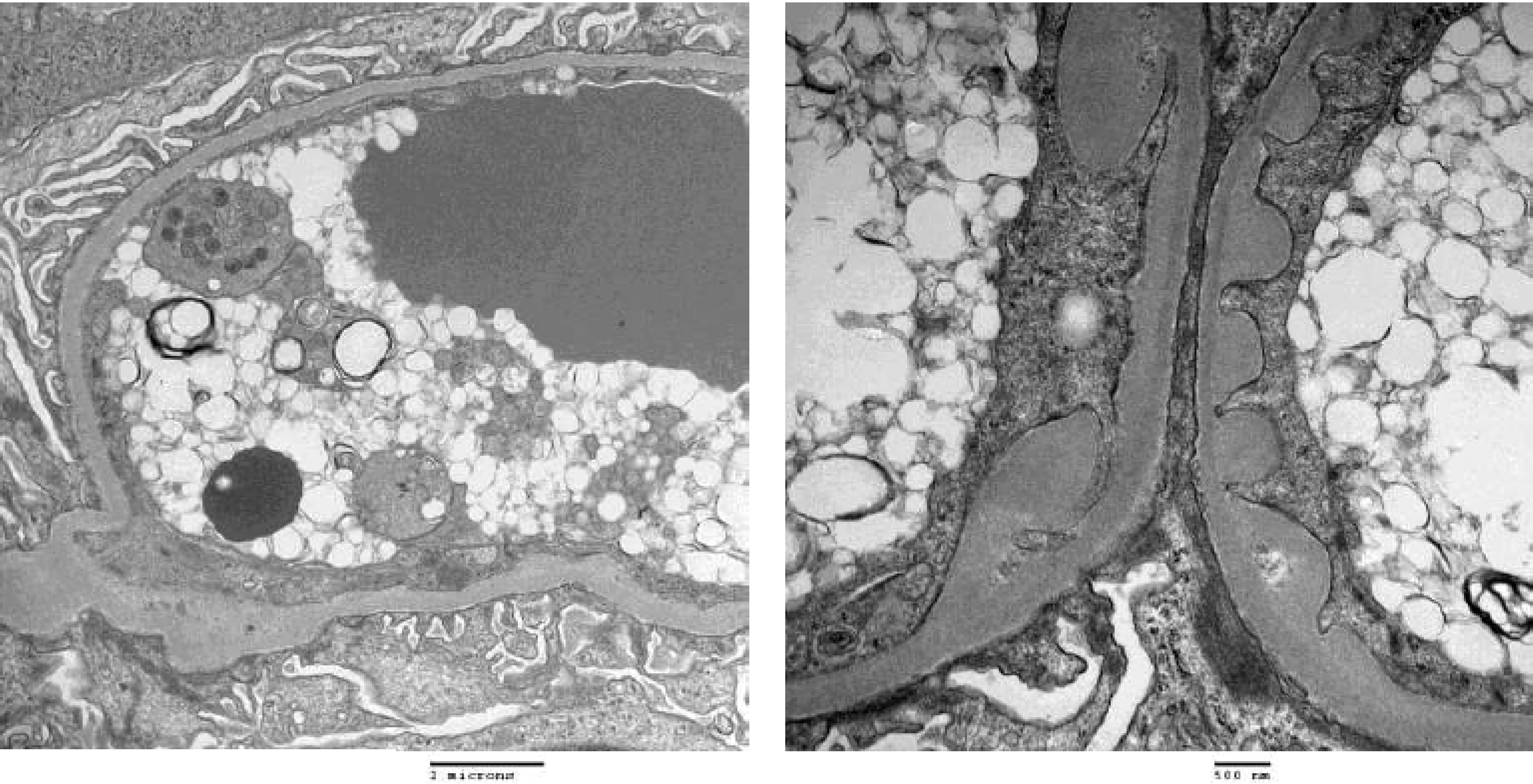

S15 Fig. Representative images from TEM of necropsy of Baboon 1, Group 6, SAL/PFC12. Liver is shown.

NECROPSY RESULTS: Liver TEM

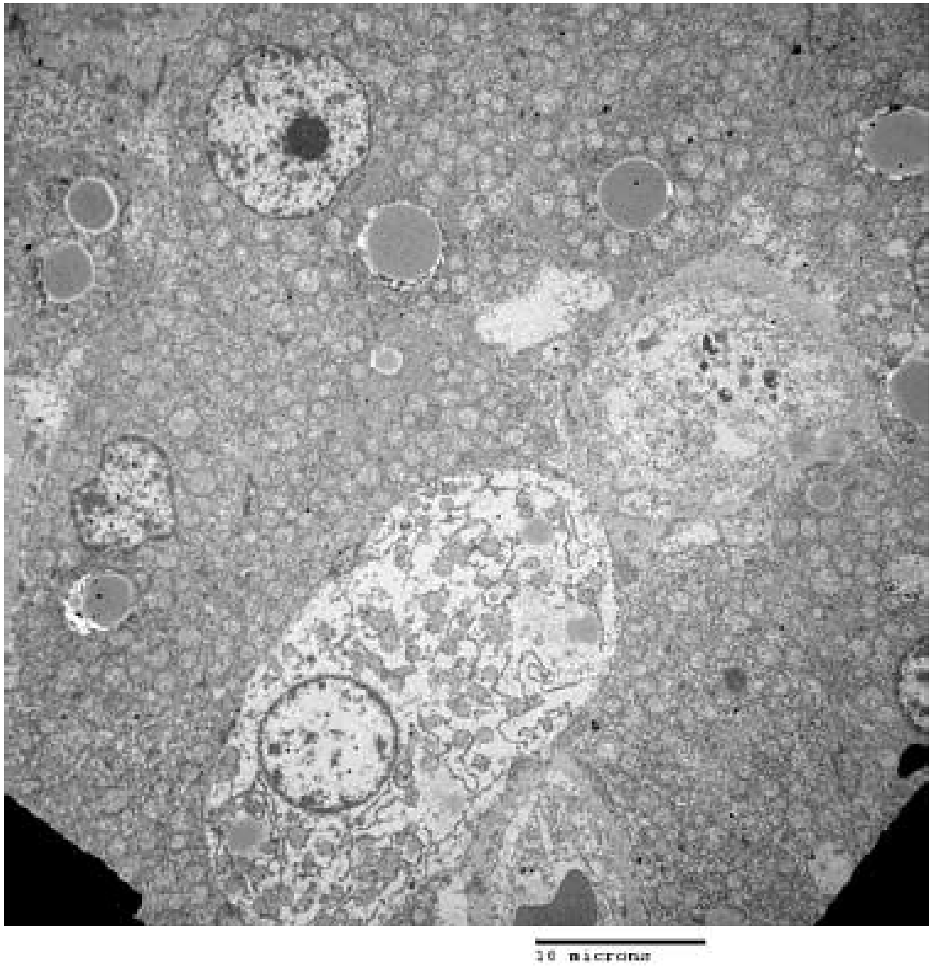

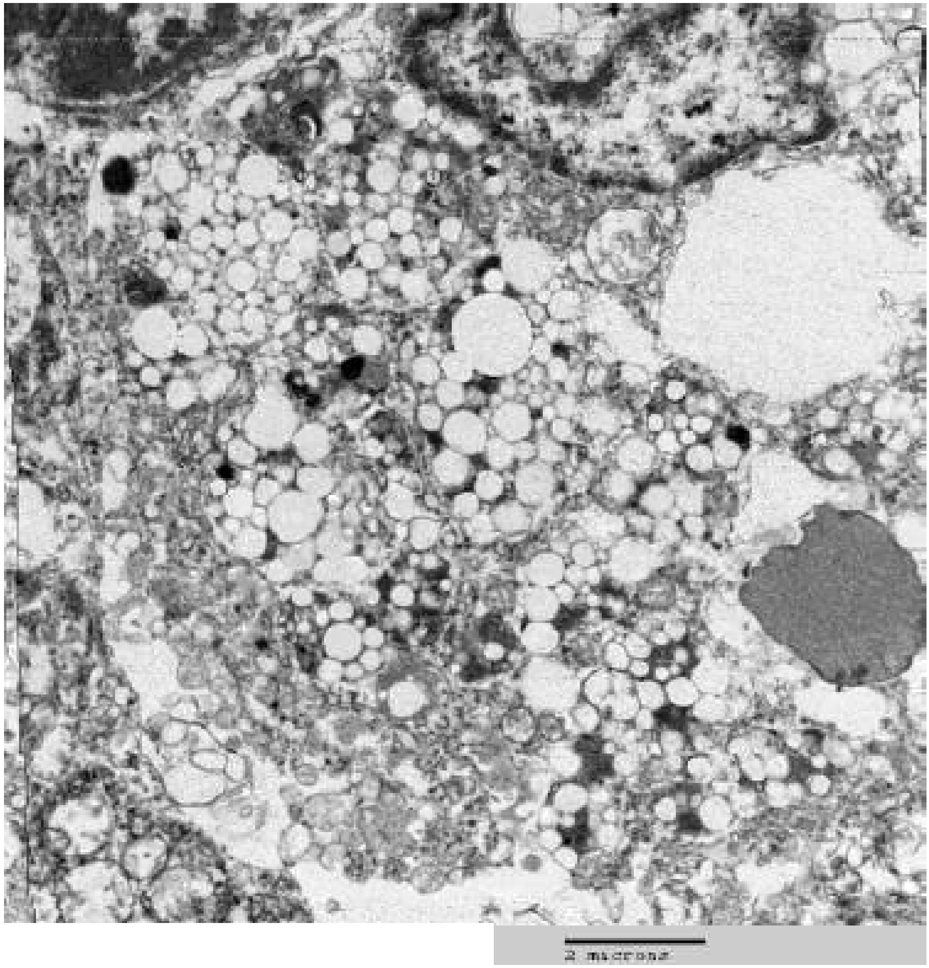

S16 Fig. Representative images from H&E of necropsy of Baboon 1, Group 6, SAL/PFC12.

NECROPSY RESULTS-Histology A&E

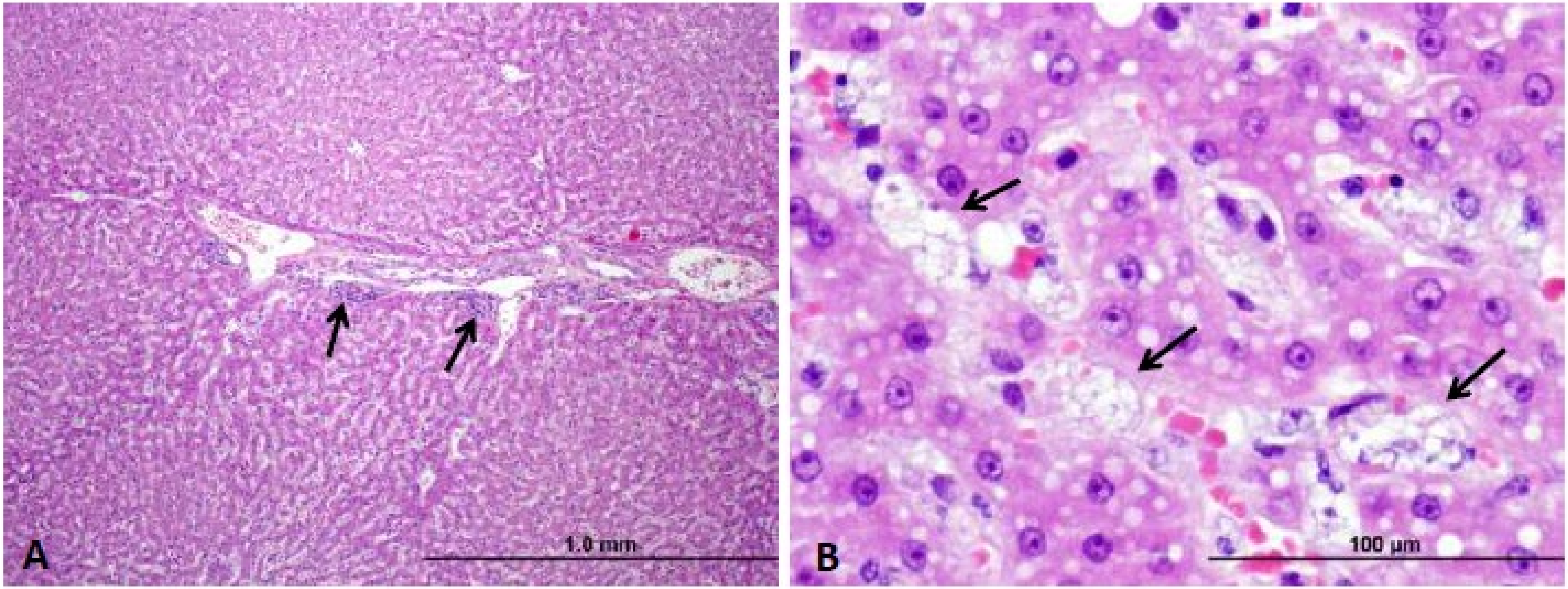

Liver:
A- Survey photograph showing dilated sinusoids and foci of chronic inflammatory cells in portal areas (arrows)
B-High magnification showing sinusoidal accumulation of foamy cells (arrows) and small lipid droplets within hepatocytes.
