# Supplementary material for: Perfluorocarbons cause thrombocytopenia, changes in RBC morphology and death in a baboon model of systemic inflammation
Source: PLoS One. 2022 Dec 30;17(12):e0279694. doi: 10.1371/journal.pone.0279694 (PMC9803179; doi:10.1371/journal.pone.0279694)
Supplement: Attachment — Submitted filename: review.docx [file pone.0279694.s003.docx]

The authors explored PFC effects on platelet function and hemostasis in a lipopolysaccharide (LPS) model of inflammation in the baboon. It was demonstrated that PFC infusion caused clinically significant thrombocytopenia and exacerbated LPS-induced platelet activation. The study was interesting and give us an explanation of the PFC caused thrombocytopenia. Yet, there are still some questions need to be answered to make the study more credible.

1.As LPS and PFC caused thrombocytopenia, platelet aggregometry should be tested using washed platelets instead of whole blood. Although the authors normalized platelet accounts.

2.From the manuscript, it’s not clarified if the effect of PFC to increase platelet aggregation was related to PFC itself or its catabolic product. So the authors need to supplement the test of PFC on platelet function in vitro in the presence of and absence of LPS. It will be much better if the authors can explore the signaling pathway changed in platelet after the incubation.

3.Representative curves of platelet aggregation should be given besides of the statistical data to make the results more visualized.

4.As the authors explained the thrombocytopenia as the increasing activation and clearance of platelet, the flow cytometry with biomarker of microphage and the classification of circulating platelets such as the constitution of pre-mature and mature platelet should be given.

5.As shown in Line 282 in the version of the article, the combination of LPS and PFC caused platelet activation similar to observations in the LPS-SAL group. Yet from Figure 4, the combination of LPS and PFC caused platelet activation much closer to observations in the SAL-SAL group. So the author need to verify the results.

6.Some of the icons in the text is not in accordance with that in the Figure, such as the Figure 7D. The authors should correct the mistakes carefully.
